# Constructive influence of the induced electron pairing on the Kondo state

**DOI:** 10.1038/srep23336

**Published:** 2016-03-24

**Authors:** T. Domański, I. Weymann, M. Barańska, G. Górski

**Affiliations:** 1 Institute of Physics, M. Curie Skłodowska University, 20-031 Lublin, Poland; 2 Faculty of Physics, A. Mickiewicz University, ul. Umultowska 85, 61-614 Poznań, Poland; 3 Institute of Physics, Polish Academy of Sciences, 02-668 Warsaw, Poland; 4 Faculty of Mathematics and Natural Sciences, University of Rzeszów, 35-310 Rzeszów, Poland

## Abstract

Superconducting order and magnetic impurities are usually detrimental to each other. We show, however, that in nanoscopic objects the induced electron pairing can have constructive influence on the Kondo effect originating from the effective screening interactions. Such situation is possible at low temperatures in the quantum dots placed between the conducting and superconducting reservoirs, where the proximity induced electron pairing cooperates with the correlations amplifying the spin-exchange potential. The emerging Abrikosov-Suhl resonance, which is observable in the Andreev conductance, can be significantly enhanced by increasing the coupling to superconducting lead. We explain this intriguing tendency within the Anderson impurity model using: the generalized Schrieffer-Wolff canonical transformation, the second order perturbative treatment of the Coulomb repulsion, and the nonperturbative numerical renormalization group calculations. We also provide hints for experimental observability of this phenomenon.

Correlated quantum impurity immersed in the Fermi sea usually develops the spin-exchange interactions^1^, that cause its total (or partial) screening below some characteristic (Kondo) temperature *T*_*K*_ 2,3. This effect is manifested in the quantum impurity spectrum by the Abrikosov-Suhl peak appearing at the Fermi level. It has been predicted^4^,^5^ and experimentally confirmed^6^,^7^ that in a correlated quantum dot (QD) embedded between metallic electrodes, such effect enhances the zero-bias tunneling conductance^8^. This situation changes, however, if one (or both) external electrode(s) is (are) superconducting because of the proximity induced electron pairing^9^,^10^. Depending on the energy level *ε*_*d*_, Coulomb potential *U*_*d*_ and the coupling Γ_*S*_ to superconducting reservoir, the ground state may evolve from the spinful configuration 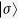 (where *σ* = ↑, ↓) to the spinless BCS-type state 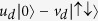
11. Such quantum phase transition (QPT) has a qualitative influence on the spin-screening mechanism^10^. In this work we show that, for Γ_*S*_ ≤ *U*_*d*_, the proximity induced electron pairing strongly amplifies the Abrikosov-Suhl peak^12^,^13^ (Fig. 1), simultaneously suppressing the QD magnetization (see Methods).

At first glance, such tendency seems to be rather counter-intuitive because Γ_*S*_ supports the proximity induced electron pairing that should compete with screening of the magnetic impurity’s spin. We provide microscopic arguments explaining this intriguing result, based on three independent methods. Our study might stimulate and guide future experimental attempts to verify this theoretical prediction in the N-QD-S heterostructures [schematically displayed in Fig. 1(a)], using e.g. self-assembled InAs quantum islands^14^, semiconducting quantum wires^15^,^16^ or carbon nanotubes^17^,^18^. Former measurements of the subgap differential conductance have already provided evidence for the Andreev/Shiba bound states^19^,^20^,^21^ and a tiny (but clear) signature of the zero-bias anomaly driven by the Kondo effect^14^,^16^,^22^,^23^. Its variation with respect to the ratio Γ_*S*_/*U*_*d*_ has not been investigated carefully enough, but this seems to be feasible.

Similar zero-bias anomalies driven by the superconducting proximity effect are nowadays intensively explored also in the quantum wires coupled to the *s*-wave superconductors, signaling the Majorana-type quasiparticles^24^,^25^,^26^. These exotic quasiparticles originate solely from the Andreev/Shiba states in the presence of the strong spin-orbit interaction and the Zeeman effect^27^. The present study might hence be useful for distinguishing the zero-bias enhancement due to the Kondo effect from the one driven by the Majorana-type quasiparticles.

## Results

In what follows we address the proximity induced electron pairing and study its feedback on the Kondo state, focusing on the deep subgap regime. First, we introduce the model and discuss its simplified version relevant for the deep subgap states. Next, we discuss the issue of singlet-doublet quantum phase transition in the limit of negligible coupling to the normal lead, Γ_*N*_ → 0, emphasizing its implications for the Kondo-type correlations. We then determine the effective spin exchange potential, generalizing the Schrieffer-Wolff transformation^1^ for the proximized quantum dot, and confront the estimated Kondo temperature with the nonperturbative NRG data (showing excellent quantitative agreement over the region Γ_*S*_ ≤ 0.9 *U*_*d*_). We also discuss the results obtained from the second-order perturbation theory (SOPT) with respect to the Coulomb potential, that provide an independent evidence for the Kondo temperature enhancement by increasing Γ_*S*_ (in the doublet state). Finally, we discuss the experimentally measurable conductance for the subgap regime and give a summary of our results.

### Microscopic model in the subgap regime

For the description of the N-QD-S junction we use the Anderson impurity model^28^


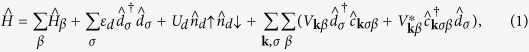


where *β* refers to the normal (*β* = *N*) and superconducting (*β* = *S*) electrodes, respectively. The operator 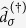 annihilates (creates) an electron with spin *σ* and energy *ε*_*d*_ in the quantum dot, while *V*_**k***β*_ denotes the tunneling matrix elements. The repulsive Coulomb potential is denoted by *U*_*d*_ and 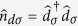. Itinerant electrons of the metallic reservoir are treated as free fermions, 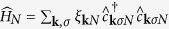, and the isotropic superconductor is described by the BCS model 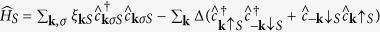. Here, 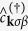 denotes the annihilation (creation) operator of a spin-*σ* electron with momentum **k** and energy *ξ*_**k***β*_ in the lead *β*, while Δ denotes the superconducting energy gap. It is convenient to introduce the characteristic couplings 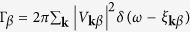, assuming that they are constant within the subgap energy regime 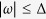.

Since we are interested in a relationship between the Andreev/Shiba quasiparticles and the Kondo state we can simplify the considerations by restricting ourselves to an equivalent Hamiltonian^29^


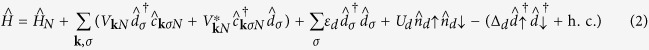


relevant for the subgap regime in a weak coupling limit Γ_*S*_ < Δ. Effects due to the superconducting electrode are here played by the induced on-dot pairing gap Δ_*d*_ = Γ_*S*_/2 9,11. This Hamiltonian (2) neglects the high-energy states existing outside the energy gap window 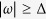 (see Methods) that are irrelevant for the present context.

### Subgap quasiparticles of the proximized quantum dot

To understand the influence of electron pairing on the Kondo effect, it is useful to recall basic aspects of the singlet-doublet quantum phase transition in the ‘superconducting atomic limit’ Γ_*N*_ → 0 9,30. Exact eigenstates of the proximized QD are then represented either by the spinful configurations 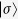 with eigenenergy *ε*_*d*_, or the spinless (BCS-type) states


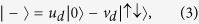



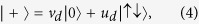


whose eigenvalues are


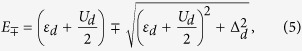


with the BCS coefficients


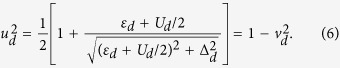


The single particle excitations, between the doublet and singlet configurations, give rise to the following quasiparticle branches ±*U*_*d*_/2 ± *E*_*d*_, where 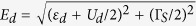. Two energies ±(*U*_*d*_/2 − *E*_*d*_) can be regarded as the low-energy excitations, whereas the other ones (shifted from them by *U*_*d*_) represent the high-energy features. In realistic systems (where *U*_*d*_ is typically much larger than Δ) the latter ones usually coincide with a continuum formed outside the subgap regime^14^,^15^,^16^,^17^,^18^,^31^.

Diagonal part of the single particle Green’s function (for its definition see Methods) is in the subgap regime given by^11^


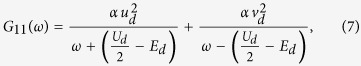


where the partial spectral weight is 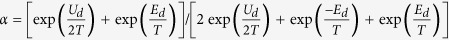 and we set the Boltzmann constant equal to unity, *k*_*B*_ ≡ 1. The missing amount of the spectral weight 1 − *α* belongs to the high-energy states existing outside the superconductor gap. At zero temperature, the subgap weight changes abruptly from *α* = 0.5 (when *E*_*d*_ < *U*_*d*_/2) to *α* = 1 (when *E*_*d*_ > *U*_*d*_/2). At *E*_*d*_ = *U*_*d*_/2 the quasiparticle crossing is a signature of the quantum phase transition from the doublet 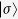 to the singlet configuration 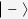
9,11,13.

**Figure 2:**
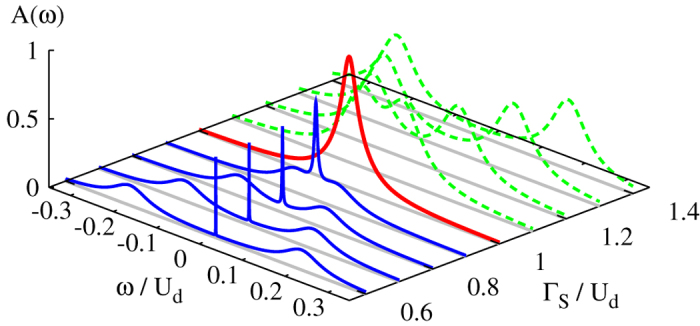
Subgap spectrum. The normalized spectral function 
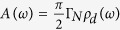
 of the half-filled quantum dot obtained from the superconducting atomic limit solution (using the quasiparticle broadening Γ_*N*_ = 10^−1^ Γ_*S*_) superposed with the Abrikosov-Suhl peak whose half width *T*_*K*_ is expressed by Eq. (21). The solid/dashed lines correspond to the doublet/singlet ground state configuration and the thick-red curve indicates the quantum phase transition at Γ_*S*_ = *U*_*d*_.

For infinitesimally small coupling Γ_*N*_ one can extend the atomic limit solution (7) by imposing the quasiparticle broadening 
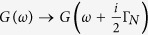
. Figure 2 shows the normalized spectral function 
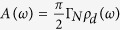
, with *ρ*_*d*_(*ω*) ≡ −*π*^−1^ Im*G*_11_(*ω*), for the half-filled quantum dot, *ε*_*d*_ = −*U*_*d*_/2. On top of these curves we have added the Abrikosov-Suhl peak (at *ω* = 0) whose half width is given by the Kondo temperature, see Eq. (21). Upon increasing the ratio Γ_*S*_/*U*_*d*_, the Andreev quasiparticle peaks move closer and they ultimately merge at the critical point Γ_*S*_ = *U*_*d*_, and simultaneously the Abrikosov-Suhl peak gradually broadens all the way up to the QPT. For Γ_*S*_ > *U*_*d*_, the Andreev peaks drift away from each other (see the dashed lines in Fig. 2) and the Kondo feature disappears for the reasons discussed in the next subsection.

### Spin exchange interactions and Kondo temperature

Adopting the Schrieffer and Wolff approach^1^ to the Hamiltonian (2) of the proximized quantum dot we can design the canonical transformation


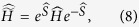


which perturbatively eliminates the hybridization term 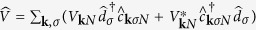. To simplify the notation, we skip the subindex *N* that unambiguously refers to the metallic lead. The terms linear in *V*_**k***N*_ can be cancelled in the transformed Hamiltonian 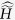 by choosing the operator 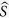 from the following constraint


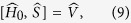


where 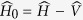. For the Hamiltonian (2) this can be satisfied with the anti-hermitian operator 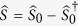, where


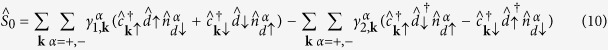


and^1^


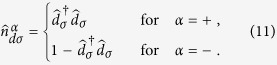


The second term of Eq. (10) explicitly differs from the standard operator used by Schrieffer and Wolff^1^. From the lengthy by straightforward algebra we find that the constraint (9) implies the following coefficients 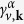



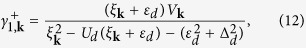



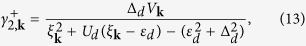



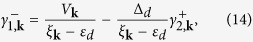



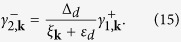


For Δ_*d*_ = 0, the coefficients 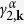 identically vanish and the other ones, given by Eqs (12 and 14), simplify to the standard expressions 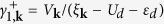 and 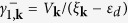 of the Schrieffer-Wolff transformation^1^.

In the transformed Hamiltonian


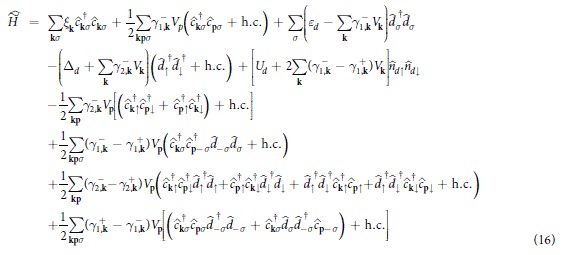


we can recognize: the spin exchange term, the interaction between QD and itinerant electrons, the pair hopping term, and renormalization of the QD energy and the on-dot pairing. Since we focus on the screening effects, we study in detail only the effective spin-exchange term


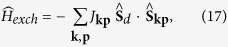


where 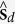 describes the spin operator of the dot and 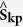 refers to the spins of itinerant electrons in metallic lead. Other contributions are irrelevant for the Kondo physics.

Formal expression for the effective exchange potential


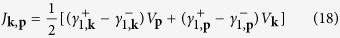


is analogous to the standard Schrieffer-Wolff result^1^, but here we have different coefficients 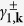 expressed in Eqs (12 and 14). This important aspect generalizes the Schrieffer-Wolf potential^1^ and captures the effects induced by the on-dot pairing.

In particular, near the Fermi momentum the exchange potential (18) simplifies to


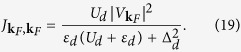


It is worthwhile to emphasize that this formula (19) precisely reproduces constraint for the quantum phase transition discussed in the previous section. To prove it, we remark that 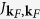 changes discontinuously from the negative (antiferromagnetic) to the positive (ferromagnetic) values at 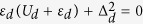. Such changeover occurs thus at


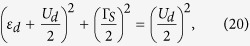


which is identical to the QPT constraint 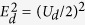 originally derived in ref. 11.

To estimate the effective Kondo temperature in the case of spinful configuration (for Γ_*S*_ < *U*_*d*_), we use the formula^32^,^33^, 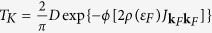, where *ρ*(*ε*_*F*_) is the density of states at the Fermi level, *D* is the cut-off energy and the auxiliary function is defined as 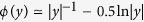. In present case the Kondo temperature is expressed by


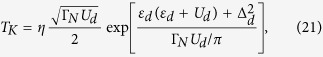


with *η* being a constant of the order of unity. Influence of the on-dot pairing on the Kondo temperature can be well illustrated considering the half-filled quantum dot case *ε*_*d*_ = −*U*_*d*_/2. The spin exchange potential (19) is then given by


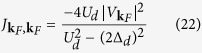


and for Δ_*d*_ = 0 it reproduces the standard Schrieffer-Wolff result^1^


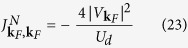


characteristic for the impurity hosted in the metallic reservoir. The relative change of 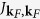 arising from the on-dot pairing is


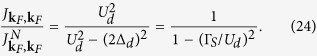


For the doublet phase (Γ_*S*_ < *U*_*d*_) the exchange coupling is antiferromagnetic, whereas for the singlet state (Γ_*S*_ < *U*_*d*_) it becomes ferromagnetic. In the latter case, however, such ferromagnetic interactions are ineffective because the spinless BCS singlet, 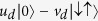, cannot be screened.

**Figure 3:**
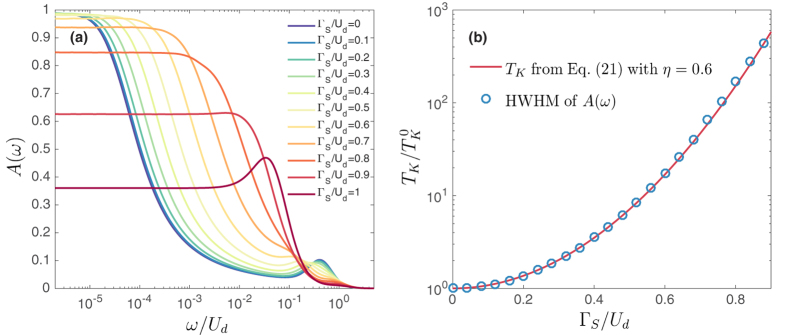
Kondo temperature. (**a**) The normalized spectral function *A*(*ω*) of the correlated quantum dot obtained by NRG for the model Hamiltonian (2) at half-filling for different values of Γ_*S*_, as indicated. Note the logarithmic scale for energies. (**b**) The Kondo temperature *T*_*K*_ extracted from the half width at half maximum (HWHM) of the Abrikosov-Suhl peak (circles) and *T*_*K*_ obtained from Eq. (21) with *η* = 0.6 (solid line). 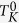 denotes the Kondo temperature in the case of Γ_*S*_ = 0, 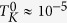. The parameters are: *U*_*d*_ = 0.1 and Γ_*N*_ = 0.01. All parameters are in units of band halfwidth *W* ≡ 1.

The estimated Kondo temperature (21) increases versus Γ_*S*_, all the way to the critical point at Γ_*S*_ = *U*_*d*_. Such tendency, indicated previously by the NRG data^13^, is solely caused by the quantum phase transition. In a vicinity of the QPT the divergent exchange coupling (22) is a typical drawback of the perturbative scheme. Figure 3 demonstrates that the formula (21) is reliable over the broad regime Γ_*S*_ ≤ 0.9 *U*_*d*_. This straightforward conclusion can be practically used by experimentalists.

### Equilibrium transport properties

We now corroborate the analytical results with accurate numerical renormalization group calculations^34^,^35^. In NRG, the logarithmically-discretized conduction band is mapped onto a tight binding Hamiltonian with exponentially decaying hopping, 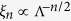, where Λ is the discretization parameter and *n* site index. This Hamiltonian is diagonalized in an iterative fashion and its eigenspectrum is then used to calculate relevant expectation values and correlation functions. In our calculations, we assumed Λ = 2 and kept *N*_*k*_ = 2048 states during iteration exploiting Abelian symmetry for the total spin *z*th component^36^. Moreover, to increase accuracy of the spectral data we averaged over *N*_*z*_ = 4 different discretizations^37^,^38^. We also assumed flat density of states, *ρ* = 1/2 *W*, with *W* the band half-width used as energy unit *W* ≡ 1, *U*_*d*_ = 0.1, Γ_*N*_ = 0.01 and zero temperature. In the absence of superconducting correlations, Γ_*S*_ = 0, this yields the Kondo temperature, 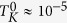, obtained from the half width at half maximum (HWHM) of the dot spectral function *ρ*_*d*_(*ω*) calculated by NRG.

Figure 3(a) presents the energy dependence of the normalized spectral function *A*(*ω*) of the correlated quantum dot at half-filling for the model Hamiltonian (2) calculated for different values of Γ_*S*_. In the case of Γ_*S*_ = 0, *A*(*ω*) exhibits Hubbard resonance for *ω* = ±*U*_*d*_/2 and the Abrikosov-Suhl peak at the Fermi energy, *ω* = 0. It is clearly visible that increasing Γ_*S*_ leads to the broadening of the Abrikosov-Suhl peak. In Fig. 3(b) we compare the relative change of the Kondo temperature obtained from the HWHM of *A*(*ω*) calculated by NRG (circles) and from the approximate formula (21) based on the generalized Schrieffer-Wolff canonical transformation (solid line). The numerical constant *η* was estimated to be *η* = 0.6. The agreement is indeed very good and small deviations occur only close to Γ_*S*_ = *U*_*d*_, but then the system is no longer in the local moment regime and the Kondo effect disappears.

**Figure 4:**
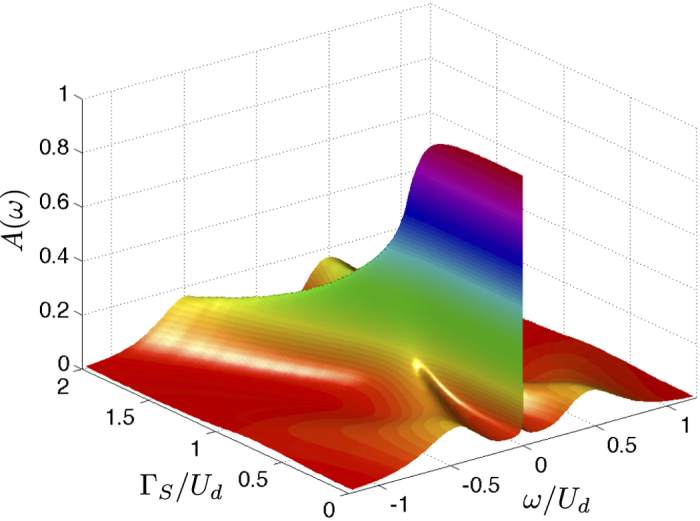
Changeover of the subgap spectrum. The normalized spectral function *A*(*ω*) of correlated quantum dot obtained by NRG calculations for 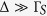 plotted as a function of energy *ω* and Γ_*S*_. The Abrikosov-Suhl peak is present in the doublet region, Γ_*S*_ < *U*_*d*_, while in the singlet region, Γ_*S*_ > *U*_*d*_, the Abrikosov-Suhl peak no longer exists. The parameters are the same as in Fig. 3.

The normalized spectral function of the half-filled quantum dot in both the doublet, Γ_*S*_ < *U*_*d*_, and singlet region, Γ_*S*_ > *U*_*d*_, is shown in Fig. 4. In the doublet region we clearly observe the zero-energy Abrikosov-Suhl peak, whose width gradually increases upon increasing Γ_*S*_. Simultaneously the Andreev peak (whose width is roughly proportional to Γ_*N*_) moves toward the gap center. In the singlet state, on the other hand, the Abrikosov-Suhl peak does no longer exist and the Andreev peaks gradually depart from each other for increasing Γ_*S*_. The same evolution of the Andreev and the Abrikosov-Suhl quasiparticle peaks is illustrated in Fig. 2, combining the superconducting atomic limit solution with the perturbative estimation of the Kondo temperature (21).

Broadening of the Abrikosov-Suhl peak upon approaching the doublet-singlet transition can be independently supported by the second-order perturbative treatment of the Coulomb interaction term 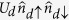. The first- and second-order contributions have been discussed in the context of Andreev^39^,^40^ and Josephson spectroscopies^41^,^42^,^43^. Here we focus on the Kondo effect, studying its evolution near Γ_*S*_ ~ *U*_*d*_. Diagonal and off-diagonal parts of the self-energy can be expressed by^40^


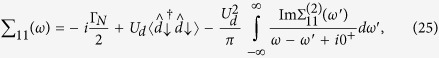



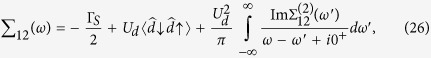


with


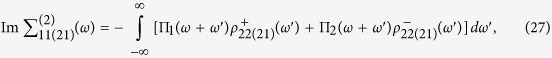



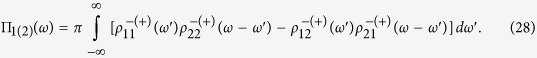


In equations (27 and 28) we have introduced 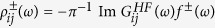, where 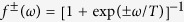 denotes the particle/hole Fermi-Dirac distribution function and 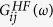 is the Green’s function obtained at the Hartree-Fock level 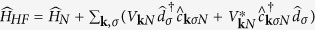 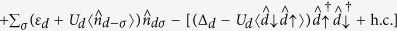. When calculating the convolutions (27,28) we have used the identities 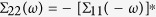 and 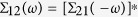.

**Figure 5:**
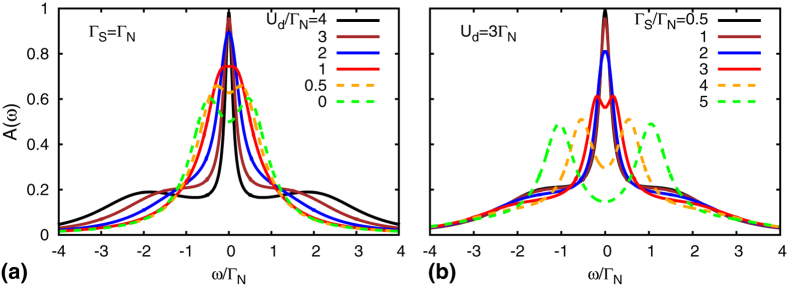
Subgap spectrum determined by the SOPT method. (**a**) The normalized spectral function *A*(*ω*) at half-filling and *T* = 0 obtained from the SOPT calculations for different values of the Coulomb correlation parameter and Γ_*S*_ = Γ_*N*_. (**b**) The same spectrum obtained for different ratios of Γ_*S*_/Γ_*N*_ with *U*_*d*_ = 3Γ_*N*_.

Figure 5 shows the spectral function *A*(*ω*) obtained from the numerical self-consistent solution of Eqs (25, 26, 27, 28). For comparison with the NRG results we focused on the half-filled quantum dot 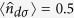. In the weakly correlated case *U*_*d*_ ≤ Γ_*S*_ (corresponding to the spinless BCS-type ground state) the subgap spectrum is characterized by two Andreev states (shown by the dashed-line curves). For *U*_*d*_ ~ Γ_*S*_, these Andreev states merge, forming a broad structure around the zero energy. In the strongly correlated case *U*_*d*_ ≥ Γ_*S*_ (corresponding to the spinful doublet configuration) we observe appearance of the Kondo feature (at zero energy) that coexists with the Andreev states^44^. We also notice that the width of the zero-energy peak (i.e. 2*T*_*K*_) depends on the ratio *U*_*d*_/Γ_*S*_ and such tendency qualitatively agrees with our estimations based on the Schrieffer-Wolff transformation and with the nonperturbative NRG data.

### Differential Andreev conductance

We now analyze how the observed features reveal in the nonlinear response regime. For possible correspondence with the experimentally measurable quantities we consider the subgap Andreev current


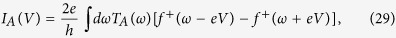


driven by the applied bias voltage *V*. The Andreev transmittance depends on the off-diagonal (anomalous) Green’s function 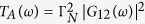. We have computed the differential conductance 
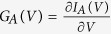
 determining the non-equilibrium transmittance by the technique described in Methods.

**Figure 6:**
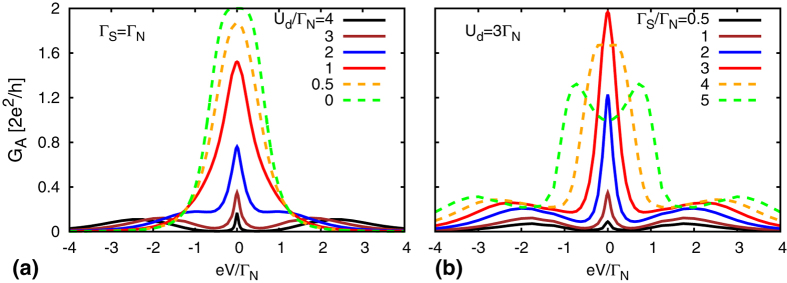
Andreev conductance. The bias voltage dependence of the differential conductance *G*_*A*_ obtained from the SOPT calculations for the half-filled QD at *T* = 0. (**a**) The conductance *G*_*A*_ for different values of the Coulomb potential *U*_*d*_ and Γ_*S*_ = Γ_*N*_. (**b**) The subgap conductance for different values of the coupling to superconducting lead Γ_*S*_ and for *U*_*d*_ = 3Γ_*N*_. We notice that the zero-bias feature induced by the Kondo effect is present only for Γ_*S*_ < *U*_*d*_ (in the spinful doublet) and its width gradually broadens with increasing Γ_*S*_/*U*_*d*_.

Figure 6 shows the qualitative changeover of the subgap conductance for representative values of *U*_*d*_ and Γ_*S*_, corresponding to doublet and singlet states. While approaching the QPT from the doublet side, we observe that the zero-bias Abrikosov-Suhl peak is gradually enhanced, and its width significantly broadens. This tendency is caused by the characteristic Kondo temperature, which increases with increasing Γ_*S*_/*U*_*d*_. For Γ_*S*_ > *U*_*d*_, however, the Kondo feature is completely absent (in agreement with NRG and Schrieffer-Wolff estimations). The magnitude of the subgap Andreev conductance approaches then the maximum value 4*e*^2^/*h* near the Andreev/Shiba states. We notice the quantitative difference between the subgap transport properties (shown in Fig. 6) and the electronic spectrum (displayed in Figs 4 and 5). Observability of the Kondo enhancement would be thus possible only close to the QPT on the doublet side.

## Discussion

We have studied the influence of the electron pairing on the Kondo effect in the strongly correlated quantum dot coupled (by Γ_*N*_) to the metallic and (by Γ_*S*_) to superconducting reservoirs by three independent methods. The proximity induced on-dot pairing and the Coulomb repulsion *U*_*d*_ are responsible for the quantum phase transition between the (spinless) BCS-like singlet and the (spinful) doublet configurations, depending on the ratio of Γ_*S*_/*U*_*d*_. Upon approaching this quantum critical point from the doublet side, one observes the enhancement of the Kondo temperature with increasing Γ_*S*_ 13. We have provided the microscopic arguments supporting this behavior based on the generalized Schrieffer-Wolff canonical transformation. This perturbative treatment of the coupling to metallic lead revealed enhancement of the antiferromagnetic spin-exchange potential, responsible for the Abrikosov-Suhl resonance. We have compared the estimated Kondo temperature with the numerical renormalization group calculations, and found excellent agreement over the broad regime Γ_*S*_ < 0.9 *U*_*d*_. We have confirmed this tendency (for arbitrary Γ_*N*_) using the second-order perturbative treatment of the Coulomb interaction. Our analytical estimation of the Kondo temperature (21) can be quantitatively verified in experimental measurements of the differential Andreev conductance. We have shown, that the zero-bias enhancement of the subgap conductance (already reported^14^,^16^,^22^,^23^ for some fixed values of Γ_*S*_) would be significantly amplified with increasing the ratio Γ_*S*_/*U*_*d*_, but only on the doublet side. Such behaviour is in stark contrast with the zero-bias anomaly caused by the Majorana quasiparticles due to the topologically non-trivial superconductivity.

## Methods

### The deep subgap regime |w| ≪ ▵

When studying the proximity effect of the Anderson-type Hamiltonian (1) one has to consider the mixed particle and hole degrees of freedom. This can be done, by defining the matrix Green’s function 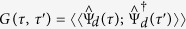 in the Nambu spinor representation, 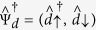, 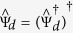. Here we determine its diagonal and off-diagonal parts in the equilibrium case (which is also useful for description of the transport within the Landauer formalism). The Fourier transform of the Green’s function 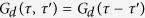 can be expressed by the Dyson equation


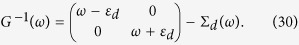


The self-energy 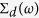 accounts for the coupling of the quantum dot to external reservoirs and for the correlation effects originating from the Coulomb repulsion *U*_*d*_.

The quantum dot hybridization with the leads can be expressed analytically by 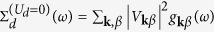, where *g*_**k***β*_(*ω*) are the (Nambu) Greens’ functions of itinerant electrons. In the wide-band limit this self-energy is given by the following explicit formula^11^,^40^


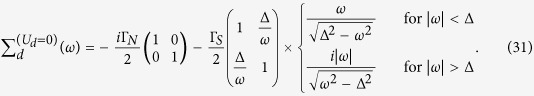


Equation (31) describes: (i) the proximity induced on-dot pairing (via the term proportional to Γ_*S*_) and (ii) the broadening (finite life-time) effects. The latter come from the imaginary parts of self-energy (31) and depend either on both couplings Γ_*β*=*N*,*S*_ (for energies 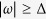) or solely on Γ_*N*_ (in the subgap regime 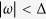).

**Figure 7:**
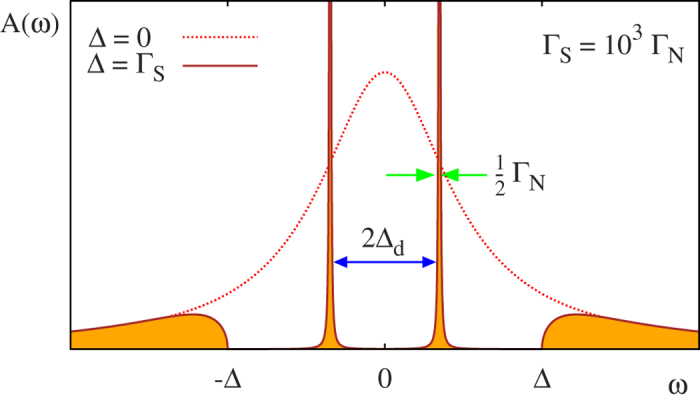
Andreev bound states of the uncorrelated QD. Spectral function *A*(*ω*) of the uncorrelated QD obtained for *ε*_*d*_ = 0, Γ_*N*_ = 10^−3^ and Δ = Γ_*S*_. The dashed line shows the reference spectrum in the absence of superconducting reservoir, Δ = 0. The in-gap states are separated by 2Δ_*d*_.

In the subgap regime 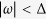 the Green’s function of uncorrelated quantum dot acquires the BCS-type structure


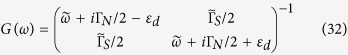


with 
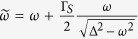
 and 
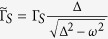
. The resulting spectrum consists of two in-gap peaks, known as the Andreev^11^,^19^ or Yu-Shiba-Rusinov^20^,^21^ quasiparticles. Their splitting is a measure of the pairing gap Δ_*d*_ induced in the quantum dot. Figure 7 displays the characteristic energy scales of the uncorrelated quantum dot.

For infinitesimally weak coupling Γ_*N*_ = 0^+^ the in-gap states have a shape of Dirac delta functions (i.e. represent the long-lived quasiparticles). Otherwise, they acquire a finite broadening proportional to Γ_*N*_. In the absence of correlations (for *U*_*d*_ = 0) the quasiparticle energies *E*_*A*,±_ can be determined by solving the following equation^45^,^46^


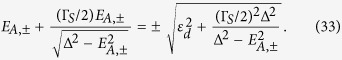


In the strong coupling limit, 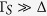, we can notice that in-gap quasiparticles appear close to the superconductor gap edges 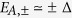, whereas in the weak coupling limit, 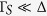, they approach the asymptotic values, 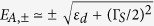. For Γ_*N*_ → 0, the latter case is known as the ‘superconducting atomic limit’. The self-energy (31) simplifies then to the static value


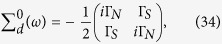


therefore the Hamiltonian (1) can be formally modeled by its fully equivalent form (2), describing the proximized quantum dot coupled to the metallic lead.

### Influence of the correlation effects

We note that the early studies of the nontrivial relationship between the Coulomb repulsion and the proximity induced electron pairing of the normal metal - quantum dot - superconductor (N-QD-S) junctions have adopted variety of the theoretical methods, such as: slave boson approach^47^,^48^, equation of motion^49^, noncrossing approximation^50^, iterative perturbation technique^39^, path integral formulation of the dynamical mean field approximation^51^, constrained slave boson method^52^, numerical renormalization group^11^,^12^,^13^,^30^, modified equation of motion^45^, functional renormalization group^53^, expansion around the superconducting atomic limit^54^, cotunneling treatment of the spinful dot^55^, numerical QMC simulations^56^, selfconsistent perturbative treatment of the Coulomb repulsion^40^ and other^9^,^46^,^57^. Amongst them only the numerical renormalization group (NRG) calculations^13^ suggested the Kondo temperature to exponetially increase with increasing Γ_*S*_ when approaching the quantum phase transition from the doublet side (Γ_*S*_ ~ *U*_*d*_).

**Figure 8:**
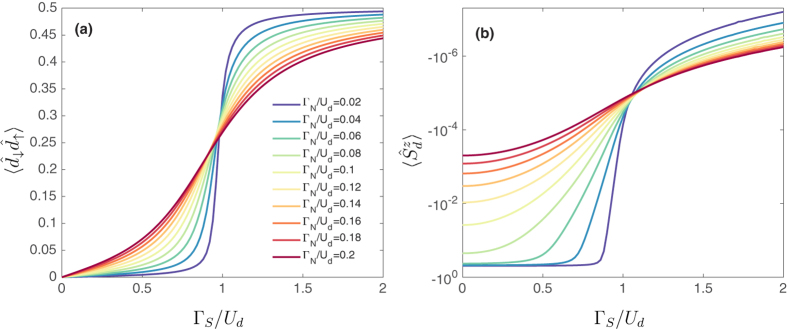
Pairing amplitude and magnetization. (**a**) The superconducting order parameter 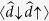 and (**b**) the magnetization 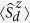 of the correlated quantum dot calculated by NRG for different couplings to normal lead Γ_*N*_, as indicated. The parameters are the same as in Fig. 3. In panel (**b**) a small external magnetic field *B* is applied to the system, *B* = 10^−6^.

The relationship between the proximity induced on-dot pairing and the screening effects can be better understood by analyzing the superconducting order parameter 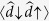 and the QD magnetization 
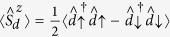
. In Fig. 8 we show their dependence on the coupling Γ_*S*_ for several Γ_*N*_/*U*_*d*_ ratios calculated by NRG. For finite superconducting energy gap a sign change of the order parameter signals the quantum phase transition^13^. However, in the case of infinite gap considered here, 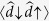 only drops to zero at the transition point^11^,^30^. As clearly seen in the figure, the order parameter 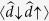 increases from 0 to 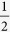 around Γ_*S*_ ~ *U*_*d*_ [Fig. 8(a)] corresponding to the QPT. Its enhancement is accompanied by the suppression of the dot magnetization, which vanishes in the singlet phase, Γ_*S*_ > *U*_*d*_, 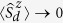 [Fig. 8(b)]. This behavior comes from the well known fact, that the local magnetic susceptibility is inversely proportional to the Kondo temperature^3^. Such variation of the QD magnetization 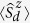 resembles the analogous tendency for the magnetic ordering in heavy fermion compounds^58^,^59^, where its suppression is driven by a competition between the local Kondo effect with the non-local RKKY interaction. In our case, the quantitative changeover of 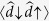 and 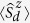 indicate the quantum phase transition at Γ_*S*_ ~ *U*_*d*_. Moreover, it can be also seen that the transitions present in the above quantities become smeared with increasing the coupling to the normal lead Γ_*N*_.

### Nonlinear charge transport in the subgap regime

Under non-equilibrium conditions the Andreev transmittance 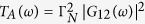 has to be determined using the lesser and greater self-energies^40^


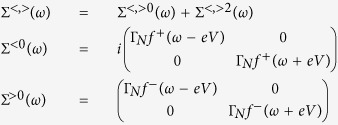


where


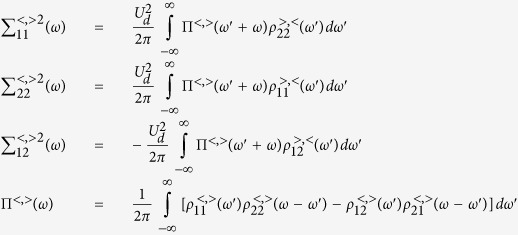


and 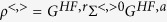, with 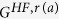 denoting the respective retarded (advanced) Green’s function. The expectation values 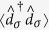 and 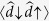 have been determined self-consistently from 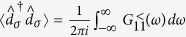 and 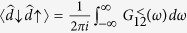, where the lesser and greater Greens’ functions obey 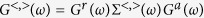, as discussed in ref. 40.

## Additional Information

**How to cite this article**: Domański, T. *et al.* Constructive influence of the induced electron pairing on the Kondo state. *Sci. Rep.* **6**, 23336; doi: 10.1038/srep23336 (2016).

## Figures and Tables

**Figure 1:**
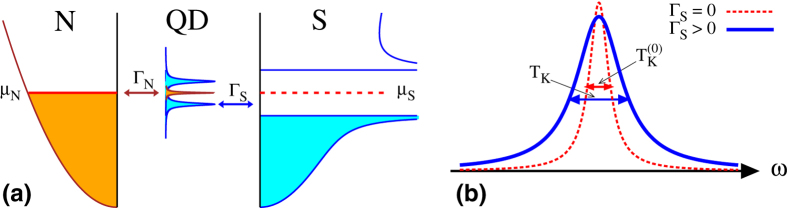
Schematic view of N-QD-S junction and the Kondo effect. (**a**) The energy spectrum in the spinful doublet configuration, where the QD Andreev bound states (driven by the coupling Γ_*S*_ to superconducting reservoir) coexist with the zero-energy Abrikosov-Suhl peak, originating from the Coulomb potential *U*_*d*_ and the coupling Γ_*N*_ to metallic lead. (**b**) Change of the width and height of the Abrikosov-Suhl resonance caused by the coupling Γ_*S*_.
